# Supplementary material for: Acute high-intensity and moderate-intensity interval exercise do not change corticospinal excitability in low fit, young adults
Source: PLoS One. 2020 Jan 24;15(1):e0227581. doi: 10.1371/journal.pone.0227581 (PMC6980578; doi:10.1371/journal.pone.0227581)
Supplement: Attachment — Submitted filename: Response to Reviewers.docx [file pone.0227581.s001.docx]

**Please see our response to comments in bold.**

Reviewer #1

Introduction

- In the introduction, I would suggest that the authors need to provide some context of the importance of neuroplasticity and why it is important / necessary to understand the effects of acute exercise on this variable. Because there is limited context provided at present, after reading the manuscript I was left wondering: ‘well, so what?’.

**To highlight the relevance of TMS in exercise research, we have added the following to the Introduction on page 3:**

**“One goal of rehabilitation is to alter corticospinal excitability, and this can be measured by changes in the MEP amplitude. Exercise is both cost-effective and can be combined with other rehabilitation protocols to augment the effects of motor re-learning [2]”**

**And on page 5:**

**“Low fit individuals are a higher risk of experiencing stroke [21]. Therefore, it is important to identify exercise regimes that increase corticospinal excitability in this population. Rehabilitation can capitalize on the post-exercise increases in corticospinal excitability, as this can prime the motor system for improvements in motor learning or re-learning [2,22].”**

- Line 59-62. The International Physical Activity Questionnaire does not necessarily indicate ‘fitness’, but instead levels physical activity. It is possible to score highly for physical activity but have a relatively poor level of cardiorespiratory fitness, and vice versa. Please consider rewriting this section (and abstract) and modifying table 1 to reflect this.

**We have modified the Abstract, Introduction and Table 1 and rewritten this line in abstract to read: “In the present study, we assessed the effect of acute high-intensity (HI) or moderate-intensity (MOD) interval exercise on neuroplasticity in individuals with low fitness, as determined by a peak oxygen uptake (VO_2peak_) test (n = 19).”**

**In the Introduction it now reads on page 3: “However, the discrepancy may also relate to the method by which fitness is assessed. For example, the International Physical Activity Questionnaire (IPAQ) is commonly used to assesses physical activity and not fitness *per se*, whereas a peak oxygen uptake (VO_2peak_) test provides an indication of cardiorespiratory fitness.”.**

- Add MEPS acronym to the key in table 1.

**The MEP acronym has now been added to the key in table 1**

- The studies reported in table 1 all appear to have used relatively small sample sizes and one wonders whether the mixed findings are in part related to low statistical power. Is this worth commenting on? I also ask the authors to provide justification of the anticipated statistical power of their own sample size in the methods, given the mixed findings in previous experiments.

**Previous studies have included sample sizes ranging from 9 to 34 participants. We based our sample size on our recent study (Lulic et al., 2017) and have included this calculation in the Methods section in the revised paper. Although we calculated 22 participants were needed in the present study, we acquired data in 19 individuals during the timeframe that was allocated to the study. After this period of time, the main experimenter (J. El-Sayes) graduated from the program and was unavailable to continue data collection. We have now added the following to the manuscript. First, in the Methods section on page 6, we include our sample size calculation that reads, “Results from Lulic et al. [6] were used to provide an estimate of the required sample size. The reported effect size for finding a change in MEPs was Cohen’s d of 0.5, and assuming a two-tailed alpha of 0.05 and power of 0.8, this yielded a sample size of 22 participants.”**

**Second, in the Limitations section on page 14, we now include the following: “Although we intended to acquire data from 22 participants, only 19 were available to us. However, a recent study from Macdonald et al. [9] observed an increase in MEPs after moderate intensity cycling in a sample of 15 participants who ranged from sedentary to fit. Therefore, it is unlikely that the lack of effect we observed is due to a limited sample size.”**

- In addition, were MEPS assessed at the same time point post-exercise in these studies? And what was the male/female balance in the study populations?

**We have modified Table 1 to include the number of females/males and the time when data were acquired. All studies had an approximately an even contribution of males and females. From the studies listed in Table 1, some previous studies assessed MEPs immediately post-exercise (Singh et al. 2014, Smith et al. 2014, Stavrinos & Coxon et al. 2017, McDonnell et al. 2013, MacDonald et al. 2019, Neva et al. 2017, Opie & Semmler et al. 2019) while others assessed MEPs at 10-15min post-exercise (Smith et al. 2014, Lulic et al. 2017, El-Sayes et al. 2019, Andrews et al. 2019) and this information is now indicated in Table 1.**

Methods

- Line 92. Please add all relevant participant characteristics here, e.g. height, mass, VO2peak etc. rather than within figure legends.

**This information has now been added to the first paragraph of the Methods on page 6:**

**“Participants had an average VO­_2peak­_ of 34.1 ± 4.0 ml/kg/min (coefficient of variation is 11.7%), height of 171.5 ± 9.5 cm, and weight of 69.9 ± 12.6 kg.”**

- Line 102. Were any criteria employed to determine if a valid maximal effort was achieved during the VO2peak test?

**Yes, as we have reported previously (Alison et al., 2017), VO_2_peak tests were considered valid if two or more of the following criteria were met: a plateau in VO_2_ despite increasing intensity, maximal heart rate within 10 bpm of age-predicted maximum, a respiratory exchange ratio > 1.1 and/or volitional exhaustion.**

**The following statement has been added to the Methods, in the “Experimental Design” section on page 7:**

**“To determine if a valid maximal effort was achieved during the VO_2_peak test, participants were required to meet two out of four of the following criteria: HR_max_ within 10 bpm of their age-predicted maximum, respiratory exchange ratio > 1.1, plateau, and/or volitional exhaustion [33]. All participants exerted maximal effort according to these criteria.”**

**Allison et al. Brief Intense Stair Climbing Improves Cardiorespiratory Fitness. Med Sci Sports Exerc, 2017.**

- Were any other controls, other than assessing physical activity, put in place prior to each experimental session? For example, nutritional standardisation, restriction of caffeine etc.? Please add.

**Yes, we screened participants for sleep deprivation, alcohol and nicotine intake 12 hours prior to each session. Further, participants were asked to refrain from any physical activity on the day of each session before arriving in our lab. The following statement has been added to the first paragraph of the Methods:**

**“Participants were asked to refrain from physical activity on the day of each session and from consuming alcohol or nicotine for 12 h prior to each session.”**

- Can the authors provide justification for why 10 minutes post-exercise is most appropriate for assessment of MEPS? This would be useful to add to the paper.

**We performed post-intervention assessments beginning at 10 minutes post-exercise to ensure the heart rate had returned to resting (baseline) levels. Further, previous studies have shown changes in corticospinal excitability when assessing MEPs in the 10-15min post-exercise (Lulic et al. 2017, El-Sayes et al. 2019). This clarification has been added to the Methods, in the “Experimental Design” section on page 7:**

**“Post-intervention assessments were obtained 10 minutes post-exercise to ensure that heart rate returned to resting levels before TMS data was collected.”**

- Similarly, can the authors comment and justify why a comparison between high and moderate intensity interval exercise was chosen as most appropriate. Moderate intensity interval exercise is not typically seen in the literature; it’s more usual to compare HIIT with moderate intensity continuous exercise.

**Had we compared a moderate intensity continuous exercise to a high intensity interval exercise, we would not be able to determine if differential effects were due to a change in exercise intensity or lack of intervals. Therefore, we chose to use a moderate intensity interval exercise to keep the modality of exercise constant. We have added the following statement to the Introduction to justify our choice of a moderate intensity interval exercise on page 5:**

**“We chose to use a moderate intensity interval exercise rather than the more commonly used continuous exercise in order to isolate the effect of exercise intensity and remove effects due to the intervallic structure of the exercise. Further, fluctuations in metabolic stress (i.e. an intermittent pattern of exercise) have been shown to influence acute skeletal muscle responses to exercise, independent of exercise intensity [25]. In addition, a recent editorial called for future research to compare physiological responses to moderate-intensity interval exercise and high-intensity interval exercise to better understand the influence of intensity independent of the exercise stimulus pattern (i.e., intermittent) [26].”**

- Given the individual differences in responses shown in figure 2, I’m curious about the reliability of this measurement techniques. Do you have data on this, e.g. coefficients of variation? Please report.

**Our laboratory has previously reported a coefficient of variation for the VO_2_peak test of 2-4% when assessed using two tests separated by ~1 wk (Burgomaster et al., 2005; Jenkins et al., 2019).**

**Burgomaster et al. Six sessions of sprint interval training increases muscle oxidative potential and cycle endurance capacity in humans. J Appl Physiol, 2005.**

**Jenkins et al. Do stair climbing exercise “snacks” improve cardiorespiratory fitness? Appl Physiol Nutr Metab, 2019.**

- Can the authors just clarify in the statistics under which circumstances they used paired t-tests or Wilxcoxon signed rank tests.
**Wilcoxon signed-rank tests were used when the data did not meet the assumption of normality. To clarify this, the following statement has been added to the Methods in the “Data Analyses” section on page 11:**

**“HI versus MOD effects on IPAQ, RPE, EMG_exercise_, and heart rate were assessed using paired t-tests in cases were data was normally distributed or Wilcoxon Signed-Rank tests in cases where data was not normally distributed.”**

Results

- Please present IPAQ data in text or in table 2.

**IPAQ data has been added to the text on page 11:**

**“Physical activity levels, assessed via IPAQ, did not differ between the two experimental sessions (HI: 2302.1 ± 2172.3; MOD: 2245.7 ± 2062.5; Wilcoxon: p = 0.65)”**

- I’m curious about the heart rate data in table 2. There appears to only very small differences in heart rate between on the lower and higher intensity intervals within each bout, much smaller than I would expect especially for HIIT. Is this because you are averaging the heart rate for each minute? If so, it may be better to report a HR during the final 10 seconds of each phase, to better reflect the fluctuations in exercise intensity. For example, based on the HR data, the MOD could be described as continuous rather than interval exercise.

**We thank the reviewer for this suggestion and yes, we initially averaged the heart rate for each minute. We have now reported the average of the final 10 seconds of both the higher (“on”) and lower (“off”) intensity intervals. This new approach has revealed even larger differences in the heart rate between the ‘on’ and ‘off’ periods.**

**These data now indicate that MOD was indeed, following an intervallic structure as confirmed by a paired t-test. We have added the following line to the Results section on page 11 to clarify that both HI and MOD were interval exercises:**

**“The average HR during the “on” and “off” intervals were significantly different in both the HI (paired t-test, p < 0.001, g = 0.85) and MOD exercises (paired t-test, p < 0.01, g = 0.41). Further, the %HR_max_ was significantly different between the “on” and “off” intervals for both the HI (paired t-test, p < 0.001, g = 0.86) and MOD exercises (paired t-test, p < 0.01, g = 0.38).” This information has also been added to Table 2.**

- I find it difficult to pick out meaningful information in figure 3C. I understand it’s purpose, but can I suggest presenting this individual response data in a different way, perhaps as individual lines overlain on a bar chart (with bars not shown), or as a dot plot with each dot representing an individual change score from pre- to post-exercise? Either of these options would make it much easier to pick out the variability in response pre- to post exercise.

**We have now replaced Figure 3C with the suggested plot.**

Discussion

- Similar to the introduction, please add some context to the discussion r.e. the importance of neuroplasticity, why it matters, and why it is important and noteworthy that you have found no effect of acute exercise in low fit individuals.

**We have added the following to the Discussion on page 14:**

**“Li et al. [22] recently showed that fast treadmill walking increases MEPs from the lesioned hemisphere in those with chronic stroke. This suggests that high-intensity exercise is a feasible method to increase motor output in stroke rehabilitation. This is in line with research showing that exercise can be used to prime the motor system to facilitate motor learning [53]. While the present study did not show an increase in motor output following high or moderate intensity interval exercise, this highlights the importance for research to determine exercise protocols that are capable of increasing motor output in this population.”**

- Line 227-229. Please consider rewriting this sentence. It is odd to say that an effect has only been observed in certain circumstances, but then follow it with ‘although not always’.

**This sentence has been rewritten as:**

**“Increases in corticospinal excitability following exercise have only been observed in high fit individuals after moderate intensity exercise [6,8,9] although some studies have reported to change after moderate intensity exercise [7,11].”**

- Please add a section on the limitations of your experiment including considerations of statistical power and reliability of measurement techniques.

**We have added the following to the limitations section in the Discussion on page 14:**

**“Although we intended to acquire data from 22 participants, only 19 were available to us. However, a recent study from MacDonald et al. [9] observed an increase in MEPs after moderate intensity cycling in a sample of 15 participants who ranged from sedentary to fit. Therefore, it is unlikely that the lack of effect we observed is due to a limited sample size”**
Reviewer #2: This study investigated the effects of two different intensities (moderate and high) of acute exercise on corticospinal excitability. The manuscript addresses a gap in the literature, which concerns the comparison of moderate and high intensity exercise in low fit individuals. The authors could neither find any changes in corticospinal excitability nor intensity dependent changes. It remains unclear why this is the case, hence, precluding any firm interpretation (e.g. interference with design issue.

Although the design fills a gap in the literature, I additionally recommend the authors to expand on the background and introduction by providing more justification for supporting the study design. For example, what is the theoretical relevance of understanding intensity differences in low fit individuals? What is the scientific underpinning leading to the current predictions?

I do have some concerns regarding the current report:


Introduction:
• It would be helpful to add a more detailed section on existing studies of varying intensity load on corticospinal excitability.

• Previous studies, which are also cited here in this manuscript, have examined low-fit subjects to investigate corticospinal excitability after acute exercise. These studies investigated low, moderate and high intensity, albeit in some cases in separate studies. The study by Smith et al also compared two exercise intensities (“low” and “moderate to high”) and could not detect any changes. Although, a direct comparison of moderate and high intensity exercise was not yet investigated, the report would be greatly improved if the derivation of the hypothesis would be described in more detail. The authors need to clarify the new insights and knowledge of their study. This is very important.
**To address the above two comments, we have now expanded the Introduction to include previous research focused on the relationship between intensity load and corticospinal excitability, highlighted the novelty of our work and clearly outlined the goals of the study on page 4:**

**“Opie & Semmler [12] reported an increase in MEPs following both low-intensity continuous and high-intensity interval cycling exercise whereas MacDonald et al. [9] demonstrated increased MEP amplitude following moderate-intensity but not low-intensity continuous cycling. In contrast, other studies have found no change in MEPs following lower versus higher intensity cycling [3,5,11]. These conflicting results demonstrate the discrepancies in the literature relating the effects of exercise intensity on corticospinal excitability. To date, no study has demonstrated changes in MEPs in low fit individuals, providing the incentive to test the effects of exercise *intensity* in this population. A direct comparison of high- versus moderate-intensity exercise is necessary to examine this question and has yet to be performed.”**

Methods:
• The authors report a study design with three sessions. I guess these were performed on separate days. It would be helpful to know the time range in which these sessions were performed (especially with regard to possible carry over effects).

**Sessions were spaced by a minimum of 48 hours. We have added this to the Methods in the “Participants” section:**

**“Nineteen individuals (22.1 ± 2.6 years; 7 females) participated in three sessions, with a minimum of 48 h between each session.”**

• Alcohol and coffein before exercising can have an influence on the physiological capacity. Did this study control for these influencing parameters?

**We asked participants to refrain from alcohol and nicotine for 12 hours prior to each session, but we did not restrict caffeine intake. We have indicated this to the Methods in the “Participants” section:**

**“Participants were asked to refrain from physical activity on the day of each session, and from consuming alcohol or nicotine for 12 h prior to each session.”**

• In the first paragraph in the methods section the authors report N=19 subjects that participated in this study. On page 6 line 113 the authors wrote: “Eight participants underwent HI in session 2 and MOD in session 3 (described below), while the other half underwent MOD followed by HI.” This suggests that only 16 subjects were included. Moreover, in Table 2 number of subjects was specified as 20. Please clarify how many subjects were included in this study/analysis.

**These were typographical errors in the original manuscript. Nineteen individuals participated in all conditions of the study. We have edited Table #2 and the text in “Experimental Design” on page 7 to read:**

**“Ten participants (3 females, 7 males) underwent HI in session 2 and MOD in session 3 (described below), while nine participants (4 females, 5 males) underwent MOD followed by HI.”**

The authors report a counterbalanced order of intervention (HI-MOD; MOD-HI) within this population. Was the order of intervention also counterbalanced between sexes?

**No, this order was not counterbalanced between sexes. We have indicated the number of females/males that received HI-MOD vs MOD-HI on page 7:
“Ten participants (3 females, 7 males) underwent HI in session 2 and MOD in session 3 (described below), while nine participants (4 females, 5 males) underwent MOD followed by HI.”**

• On Page 5, line 102/103 the authors report that the VO2peak test was performed on a cycle ergometer “Lode Excalibur Sport V2.0”. However, the interventions were performed on a different cycle ergometer “Ergo Race”. Have the authors ensured that the power of the cycle ergometers was validated/the same, e.g. through calibration?

**We have not compared the two ergometers as suggested, but this would not impact on our results. Intensity for the HI and MOD protocols was assessed based on % of maximum heart rate and not the absolute workload setting on the ergometer. Thus, any small potential differences between the two ergometers would be accounted for.**

Results:
• The authors report that the IPAQ was acquired on both examination days to ensure that a similar activity level was maintained throughout the study. Please include the IPAQ results. Was the basic activity level maintained?

**We have now included the IPAQ data in the Results on page 11. A Wilcoxon signed rank test indicated that similar activity level was maintained throughout the study:**

**“Physical activity levels, assessed via IPAQ, did not differ between the two experimental sessions (HI: 2302.1 ± 2172.3; MOD: 2245.7 ± 2062.5; Wilcoxon: p = 0.65)”**

• The authors report, that they measured the activity of the FDI muscle during exercise to ensure the muscle was inactive. Please report the results.

**These results are now reported in Table 2.**

• Results how a high variability (Figure 3C). Could this be driven by gender differences? It would make sense to exclude gender differences and to include sex as covariate in the statistical analyses.

**We do not have an even distribution or statistical power to accurately compare results between biological sex (7 females, 12 males). However, a recent study from our lab showed that biological sex does not influence exercise-induced neuroplasticity (El-Sayes et al., Neuroscience, 2019). It is more likely that the variability in the data is due to the presence of the BNDF val66met in some individuals but not others. The presence of this polymorphism has been linked to attenuation of neuroplasticity. We have added the following to the Limitations section in the Discussion on page 14:**

**“One factor that may contribute to the variability in AURC is biological sex. We did not recruit an equal ratio of male to female participants to investigate the effect of biological sex on our data. However, a recent study has reported no effect of biological sex on exercise-induced neuroplasticity. Another factor that may introduce variability is genetic variation. Those with the BDNF val66met polymorphism show reduced BDNF secretion [54] that is linked to attenuated exercise-induced neuroplasticity responses following high-intensity interval exercise [11] and motor training [55]. We did not determine the distribution of participants presenting the val66met polymorphism, and this is a limitation of the study. “**

**El-Sayes et al. The Effects of Biological Sex and Ovarian Hormones on Exercise-Induced Neuroplasticity. Neuroscience. 2019.**
